# Evaluating backscattering polarized light imaging microstructural mapping capabilities through neural tissue and analogous phantom imaging

**DOI:** 10.1117/1.JBO.29.5.052914

**Published:** 2023-12-05

**Authors:** Justina Bonaventura, Kellys Morara, Rhea Carlson, Courtney Comrie, AnneLeigh Twer, Elizabeth Hutchinson, Travis W. Sawyer

**Affiliations:** a University of Arizona, Wyant College of Optical Sciences, Tucson, Arizona, United States; b University of Arizona, Department of Biomedical Engineering, Tucson, Arizona, United States; c University of Arizona, Department of Molecular and Cellular Biology, Tucson, Arizona, United States

**Keywords:** polarized light imaging, Mueller matrix polarimetry, brain imaging, backscattering polarimetry, tissue phantoms

## Abstract

**Significance:**

Knowledge of fiber microstructure and orientation in the brain is critical for many applications. Polarized light imaging (PLI) has been shown to have potential for better understanding neural fiber microstructure and directionality due to the anisotropy in myelin sheaths surrounding nerve fibers of the brain. Continuing to advance backscattering based PLI systems could provide a valuable avenue for *in vivo* neural imaging.

**Aim:**

To assess the potential of backscattering PLI systems, the ability to resolve crossing fibers, and the sensitivity to fiber inclination and curvature are considered across different imaging wavelengths.

**Approach:**

Investigation of these areas of relative uncertainty is undergone through imaging potential phantoms alongside analogous regions of interest in fixed ferret brain samples with a five-wavelength backscattering Mueller matrix polarimeter.

**Results:**

Promising phantoms are discovered for which the retardance, diattenuation and depolarization mappings are derived from the Mueller matrix and studied to assess the sensitivity of this polarimeter configuration to fiber orientations and tissue structures.

**Conclusions:**

Rich avenues for future study include further classifying this polarimeter’s sensitivity to fiber inclination and fiber direction to accurately produce microstructural maps of neural tissue.

## 1 Introduction

Developing tools capable of visualizing brain tissue microstructure is desirable in a wide range of applications. Understanding the pathological changes in neural microstructure that occur with neurodegenerative diseases may help researchers tie those changes to biomarkers, which can then be used to develop early disease intervention strategies.^1^ Being able to clearly differentiate between regions of brain tissue, which may not be visually distinct from tissue dysplasia or cancerous tissue as a tool for surgical guidance is key to improving patient outcomes and reducing the risk of surgical complications.^2^^,^^3^ Developing a thorough understanding of the microstructural effects of traumatic brain injuries will increase the ability to model the long-term effects and help to develop better therapeutic treatments^4^^,^^5^ Overall, a more complete mapping of the neural connections and fiber tracts would be beneficial for furthering understanding of neural functioning.^6^

Polarized light imaging (PLI) is a promising method for imaging neural tissue at high resolution given its sensitivity to microstructural orientation features. In general, PLI refers to a range of imaging techniques which use polarization sensitive detectors and illumination to measure how a material interacts with polarized light. PLI has a broad range of applications in biomedical imaging as the anisotropic nature of many tissue types allow the use of PLI to acquire structural and functional information,^7^ which can be used for understanding underlying biology, elucidating disease progression, or detecting tissue abnormalities.^7^^–^^10^ PLI is particularly valuable for imaging the brain as the cellular content includes highly oriented myelin-coated axons making up white matter regions, which have a different refractive index than adjacent glia.^11^^,^^12^ The densely packed and highly ordered coherent fiber bundles have been found to have uniaxial birefringence due to the arrangements of lipids in the myelin sheath.^2^^,^^13^

PLI methods for brain imaging have been investigated for various applications. In several studies the use of PLI was considered as a tool for guiding surgical procedures including tumor resections.^2^^,^^12^^,^^14^ PLI has been shown to be useful in differentiating between healthy and cancerous tissue due to the structural changes that often result from tumor growth.^10^^,^^15^^,^^16^ In the case of neural tissue, the highly structured white matter tracts of the brain may not be visually distinct from tumors under standard white light imaging; however, if the microstructural information could be visualized, such as with PLI, contrast could be enhanced to highlight clear differences between normal tissue and chaotic tumor growth,^12^ potentially reducing the risk of damage to neurological function through excess tissue removal or incomplete resections.^2^^,^^11^ Additionally, there has been research relating the pathological effects of neurodegenerative diseases to changes in the polarization properties of neural tissue, illustrating further utility of PLI for brain imaging.^17^ While PLI is limited to surface or near-surface imaging, it also has potential to be useful in window chamber animal studies with the aim of examining the microstructural pathological effects of traumatic brain injuries and neurodegenerative diseases with reduced impact of tissue sectioning and staining.

The gold standard for *in vivo* microstructural neural imaging, diffusion magnetic resonance imaging (dMRI), is able to map the orientation of neural fibers by analyzing the anisotropic diffusion of fluid movement in the brain.^13^ However, it has some limitations such as resolution, which ranges from a few hundred micrometers to a few millimeters.^18^ This can pose challenges in resolving individual fiber characteristics, as many nerve fibers are on the order of one micrometer.^18^^–^^20^ In comparison, the PLI system used in this study has a resolution of around 10 micrometers.^21^ There are also challenges for dMRI in developing and optimizing reconstruction models for certain complex fiber arrangements, such as submillimeter crossing fibers.^18^^,^^19^ While PLI could in no way fill the role of dMRI, methods for validating dMRI measurements are desirable.^13^^,^^22^ To assess the potential of PLI as a validation technique for dMRI, several studies have shown promising results with imaging thin sectioned tissue samples in transmission mode.^23^^,^^24^ Beyond this, the feasibility and accuracy of using dMRI as an intraoperative surgical guidance tool pose challenges due to the bulky instrumentation and lengthy acquisition required to collect such images,^12^^,^^25^ for which PLI has potential to be used to complement dMRI as it is a label-free, direct method with a proven ability to probe white matter micro-structures,^7^ if it could be fully realized for *in vivo* use.

To do so, backscattering PLI methods need to be optimized for high-fidelity use on bulk tissue samples. While highly detailed three-dimensional fiber maps have been generated using transmission PLI,^6^ this requires thin sectioning of the tissue, which can introduce artifacts and eliminates *in vivo* imaging as a possibility. In addition, tissue preservation results in a departure from true *in vivo* characteristics. To date, there have been several studies, which use backscattering polarimetry to visualize white matter fiber tract direction. In fixed thick human brain and fresh calf brain samples using wide field reflection Mueller matrix polarimetery, Schucht et al. demonstrated the feasibility of using linear retardance to find the azimuth of the optic axis of the white matter tracts.^12^ Jain et al.^14^ used a polarimetric microscope to distinguish between white and gray matter and estimate white matter fiber orientation in fixed human brain samples.

One major system parameter for backscattering PLI that could benefit from further understanding is the application of imaging in multiple wavelengths. There have been several studies which observe a change in the magnitude of depolarization as the imaging wavelength changes, which are attributed to the relationship between the number of scattering events increasing as the light penetrates deeper into the tissue and wavelength specific absorption by hemoglobin.^16^^,^^26^ If optimized, this could be a valuable metric in surgery and for cancer staging as well as structural mapping, as many relevant features exist at varying depths.^12^ More rigorous studies of wavelength dependence across a broader range of polarization properties would be advantageous, in particular to understand how wavelength dictates sensitivity to structures at different depths, as well as the scale dependence of the dominant microstructural tissue components.

Another important limitation in parsing backscattering PLI data is the system response to certain fiber orientations. Among them, resolving crossing fibers has been a challenge in PLI, which has a particular relevance for brain microstructural mapping.^18^ Additionally, with three-dimensional tissue geometries—fiber orientations are generally not purely in the plane of imaging. Though the retardance response of fiber inclination is well understood for transmittance PLI,^6^^,^^13^ for backscattering PLI systems the effects have not been thoroughly investigated. Similarly, the effects of surface topology have been studied previously by Rodriguez et al. who tilted and rotated fresh calf brain samples to determine this did not diminish their ability to map the optical axis of the sample^2^ and Liu et al. who used a reflectance based polarmetric system to image various types of tumor samples and found that surface geometry had a minimal effect on distinguishing between tumor and healthy tissue.^27^ However, the effects and quantification of fibers and tissue curving out of plane on polarization properties as a whole and the ability of backscattering PLI to map those has yet to be thoroughly investigated. A better understanding of how PLI systems respond to these different microstructural arrangements is essential to advance the technique away from flat sectioned samples towards *in vivo* imaging of three-dimensional brain structures.

In working with tissue samples, it can be challenging to isolate and examine these individual properties as there are multiple confounding factors. Particularly, in regions with lower polarimetric signal, it can be difficult to parse whether there is a lack of fiber structure, the polarimeter is not sensitive to structures of that type, or there are systematic errors that produce artifacts. One way to isolate these issues to better understand how the imaging system responds to them is through imaging tissue phantoms. There have been many previous studies using PLI phantoms, reviewed in detail by Chue-Sang et al.^28^ In some cases, their main objective is to assess whether the system is able to accurately resolve polarization properties and anisotropy;^29^^–^^31^ similarly groups have used phantoms to characterize an imaging system’s responses to specific structural features;^32^ alternatively phantoms have been used to validate the mathematical formalism of decomposition methods and simulate light-tissue interactions.^33^^–^^35^ Phantom materials which have been used previously include birefringent plastic film,^29^ electrospun polymer fibers,^30^ stretched and annealed polymer samples,^36^ stretched elastic polyacrylamide polymer,^31^ extruded silicon,^32^ and well aligned silk fibers.^35^ Elements such as sucrose, microspheres, and well-aligned glass fibers have been embedded in phantoms to introduce optical activity, scattering events, and ordered anisotropy.^33^^,^^34^^,^^37^

In this paper, we expand upon work previously presented as a conference proceeding^38^ with the aim of probing several outstanding challenges in interpreting backscattering PLI results by utilizing potential PLI phantoms for these effects. With this, we present quantitative data of the effects of four different microstructural features and surface topology over five different imaging wavelengths on bulk and sectioned neural tissue as well as phantoms, allowing for a more thorough discussion of the combination of these factors than what has been previously seen for backscattering polarimetry systems. We first lay out a theoretical background of how different fiber geometries may influence measured retardance, as well as the dependence on wavelength of such measurements. Tissue phantoms are imaged using a backscattering full Mueller polarimeter to study the system’s sensitivity to various fiber configurations and geometries. For each scenario, we extract the retardance, retardance angle, diattenuation, and diattenuation angle and assess the influence of phantom geometry. We then acquire images of ferret brain samples in regions of interest (ROIs) that possess tissue fiber microstructures with similar geometries. We find that these phantoms are promising options, which begin to shed light on the ways in which this polarimetry system responds to these geometries.

## 2 Theoretical Background

In considering how retardance measurements may be effected by microstructural orientation we can model the neural tissue and phantoms as uniaxial crystals. This will induce a retardance in light transmitting through the fibers accordingly

$$\delta =\frac{2\pi \Delta nL}{\lambda_{o}},$$ (1)

where δ is the retardance, L is the path length through the material and $\lambda_{o}$ is the incident wavelength. Δn, the birefringence, is defined as

$$\Delta n(\lambda )=n_{e}(\lambda )-n_{o}(\lambda ),$$ (2)

where $n_{e}$ is the extraordinary axis of the material and $n_{o}$ is the ordinary axis.

The effects of tilting a uniaxial crystal out of plane have been derived previously.^39^^,^^40^ The effective refractive index of the extraordinary axis of the material will change with tilt accordingly

$$n_{e}(\theta_{i})=\frac{n_{O}n_{E}}{\sqrt{n_{E}^{2}\;\sin^{2}(\theta_{i})+n_{O}^{2}\;\cos^{2}(\frac{\pi }{2}-\theta_{i})}},$$ (3)

where $n_{E}$ is the refractive index of the extraordinary axis intrinsic to the material and $n_{O}$ is the ordinary and $\theta_{i}$ is the tilt angle of the optic axis of the material. This assumes the optic axis is normal to the propagation direction of incident light when not tilted. In this case, white matter fiber tracts in the brain have been found to have a negative birefringence^13^ so expect for this case $n_{E}<n_{O}$.

The polarimeter used in this study is only sensitive to the magnitude of the retardance, not the sign (positive or negative), so the effects of tilting on retardance measurements can be modeled as

$$\delta =\frac{2\pi L}{\lambda_{o}}|\frac{n_{O}n_{E}}{\sqrt{n_{E}^{2}\;\sin^{2}(\theta_{i})+n_{O}^{2}\;\cos^{2}(\theta_{i})}}-n_{O}|.$$ (4)

**Fig. 1:**
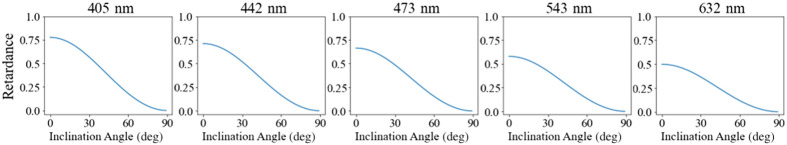
Retardance (in radians) as defined by Eq. (4) plotted versus inclination angle over five different imaging angles.

In the use of a Mueller matrix polarimeter, a full retardance Mueller matrix is derived through decomposition, from which the angle of the retardance axis of the material can be determined as the retardance angle.^33^ This can then be used to determine the direction of microstructural features, for example, the white matter fiber tracts in the brain. The polarimeter used in this study has five different imaging wavelengths; inspection of Eq. (1) indicates that we expect there to be a decrease in retardance with increased imaging wavelength, as shown in the plots in Fig. 1. There is further wavelength dependence intrinsic to the birefringence, which can be seen in Eq. (2). There have been studies that discuss wavelength-dependent birefringence in polymers, such as those which the phantoms used in this study are comprised of.^41^^,^^42^ In one such study,^41^ the change in birefringence decreased with increase in wavelength on the order of 10%. This is not fully factored into this simulation; however, it would further contribute to the effects of the wavelength dependence in Eq. (1). In considering the wavelength dependence of the neural tissue used, it is important to note that they were fixed and therefore largely free of hemoglobin, which has a known wavelength dependence in its absorption.^16^^,^^26^

The magnitude of the retardance can be simulated over the five different imaging wavelengths used in this study, as shown in Fig. 1. In our model, we assume L, the path length, is constant at all inclination angles. The path length will be defined by the mean free path of photons backscattering in the material. However, it is possible that for thin materials, the thickness of the materials is less than the mean free path. In this case, the effective path length may vary with different inclination angles. For the purposes of this theoretical model, we consider L to be wavelength independent. It is well known that light penetration depth in turbid media does vary by wavelength, so this is an approximation. To fully consider this effect, Eq. (1) would have to be adjusted such that L is wavelength dependent as proportional to the mean free path of the photons for each imaging wavelength. Estimation of this effect could be done through Monte Carlo simulations using Mie theory, as described in the literature^43^^,^^44^ or through empirical measurement. However, in general, longer wavelengths in the visible spectrum will penetrate more deeply through tissue, which would result in a possible increase in retardance, confounding the effect of the wavelength in the denominator or Eq. (4).

## 3 Methods

### 3.1 Samples Imaged

#### 3.1.1 Ferret brains

To assess the polarimeter’s sensitivity to various tissue characteristics, ferret brains used in several previous studies^45^^,^^46^ were imaged. Ferret brains were selected due to their more complex neural white matter geometries than other animals of similar size, which bear more resemblance to the structures found in human brains.^47^ The brains were removed from the ferrets postmortem after they underwent a perfusion fixation with 4% paraformaldehyde (PFA). The tissue was then post-fixed in the PFA for 10 days before being rehydrated and stored under refrigeration in a phosphate-buffered saline with 0.01% sodium azide preservative. The ferrets were all adult males around 5 to 10 months at the time of harvest and the samples have been stored for 2 to 4 years at 4°C. All animal procedures undergone in this study were approved by the University Institutional Animal Care and Use Committee, and the guidelines set by the Uniformed Services University of the Health Sciences and the National Institute of Health’s Guide for the Care and Use of Laboratory Animals were followed in the treatment of all animals used in this study.

**Fig. 2:**
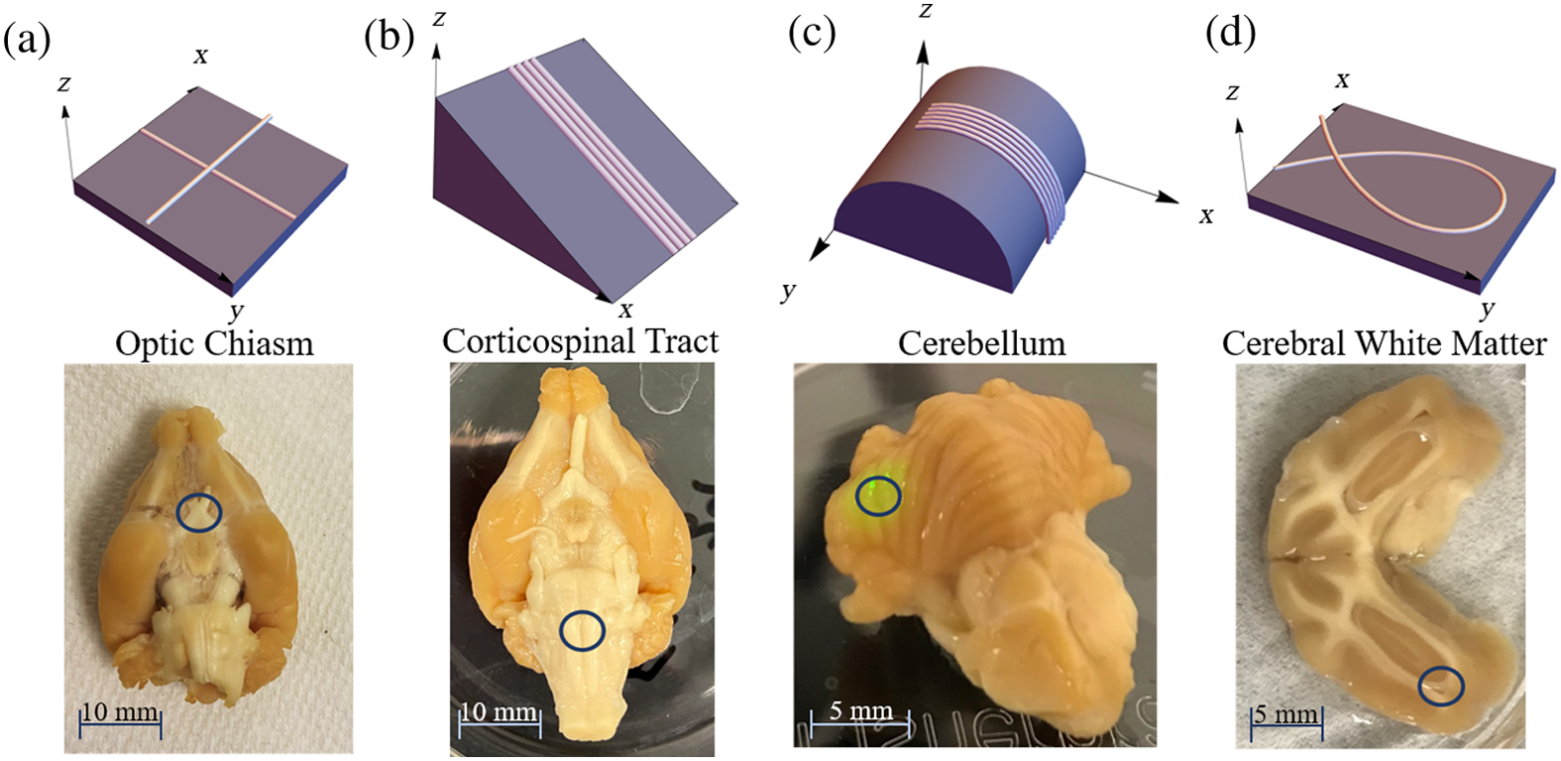
(Top row) Renderings of fiber geometries investigated in this study, and (bottom row) photographs of corresponding ferret brain ROIs. (a) Crossing fibers and optic chiasm, (b) inclined fibers and CST, (c) curving fibers out of plane and cerebellum, and (d) curving fibers in plane and cerebral white matter region.

Regions were selected to be imaged, which have known microstructural properties of interest. As shown in Fig. 2, ROIs include the optic chiasm, Fig. 2(a), due to its crossing fibers; the corticospinal tract (CST), Fig. 2(b), with distinct coherent fiber bundles primarily running in one direction; the cerebellum, Fig. 2(c), due to its three-dimensional curvature and ridges with parallel fibers running across them; and cerebral white matter, Fig. 2(d), from a sectioned sample selected for its in-plane curvature. The hippocampus was imaged as well due to its curving structure.

The samples were all imaged in bulk tissue form initially; after this, several of the samples—namely the optic chiasm and hippocampus—were paraffinized, sectioned to thin 7-micron thick sections and reimaged. This was done to access internal structures in the brain with more complex fiber geometry.

#### 3.1.2 Tissue phantoms

The tissue phantoms used in this study were thin plastic fibers, such as plastic fiber optic cables, polymer-based synthetic hair fibers from an artificial wig, and clear polyester monofilament thread (232-2001, Sulky). Using the classification scheme described by Shrivastava,^48^ the plastic fiber optic cables were determined to be polystyrene based, and the synthetic hair to be polyvinyl chloride (PVC) based. From our preliminary studies, the fiber optic cable and synthetic hair strand both have very strong and coherent retardance and retardance angle signal, and the monofilament thread has a strong and coherent diattenuation signal. These fibers were easily manipulated into the desired configurations: namely crossing fibers, curving fibers, and inclined fibers, by securing the fibers to black aluminum foil for a dark imaging background and attaching them to card stock in the correct orientation, tilting secured fibers upwards and securing the fibers over a cylindrical form to be imaged. To be sure the construction of the phantoms did not induce strain and change the value of the retardance, the fibers were secured gently within their natural radius of curvature. The fiber optic cable had a diameter of 0.29 mm with a standard deviation of 0.018 mm, the synthetic hair—0.060 mm with a standard deviation of 0.010 mm, and the monofilament thread—0.063 mm with a standard deviation of 0.0068 mm. To account for the slight variations in the fiber diameters, the same fibers were used throughout each experiment. In considering the birefringence of the different phantom types, while the manufacturing processes these fibers undergo may affect this, polystyrene fibers have been shown to be negatively birefringent in previous studies,^49^^,^^50^ and PVC has been found to be both positively and negatively birefringent in previous studies, but more commonly positively birefringent.^50^ This will provide a good comparison to white matter fiber tracts, which have been shown to be negatively birefringent.^13^

### 3.2 Data Acquisition and Processing

**Fig. 3:**
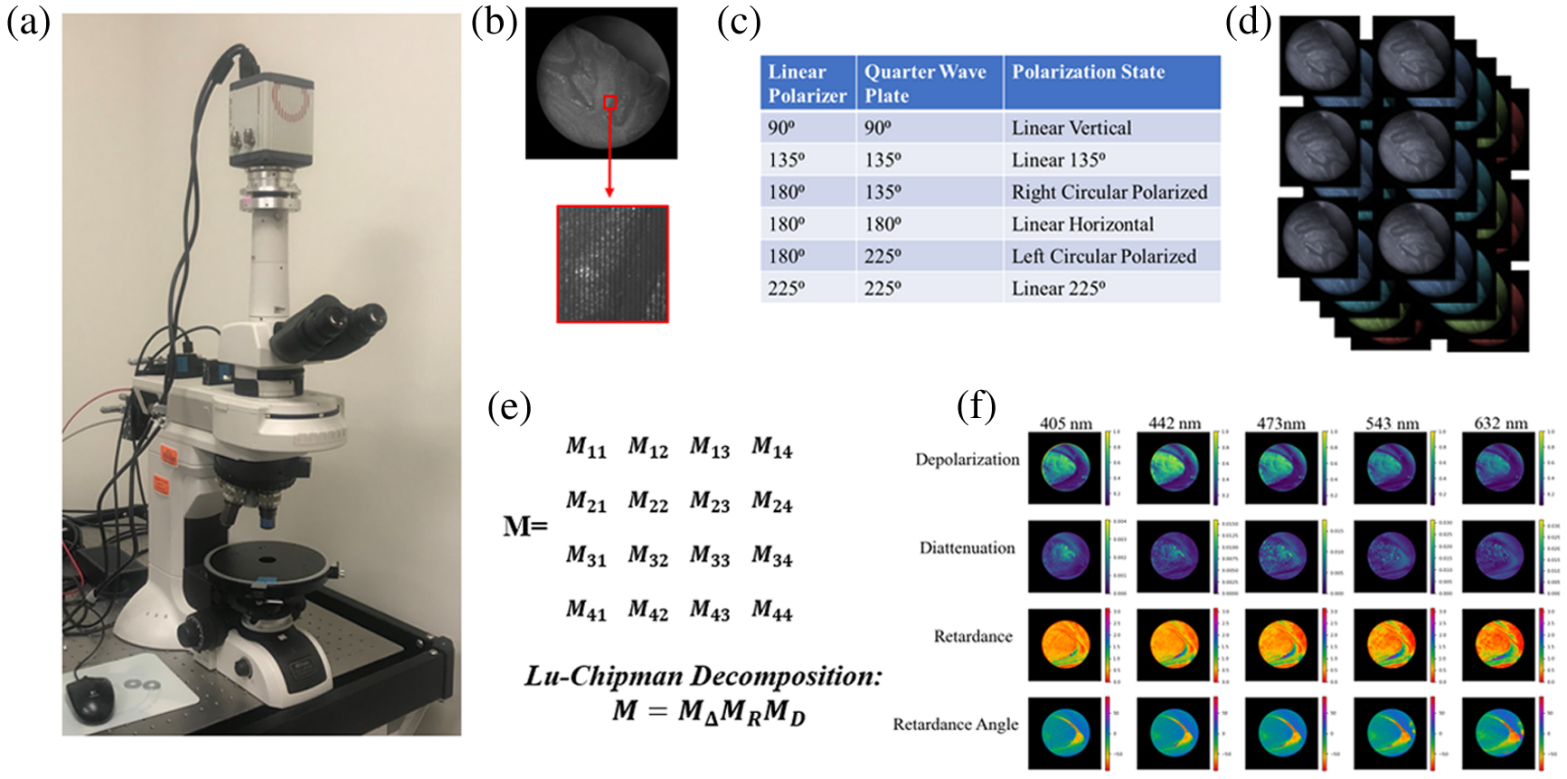
(a) Photograph of polarimetry microscope. (b) Example image from the polarimeter with detail of Stokes parameters encoded into frequencies due to Savart plate polarization state analyzer. (c) Summary of polarization states used as illumination for the system. (d) Set of six images taken for each of the five wavelengths. (e) Resulting Mueller matrix output and Lu–Chipman decomposition. (f) Decomposed pixel-wise maps of depolarization, diattenuation, retardance, and retardance angle over each of the imaging wavelengths.

The polarimeter used in this study, which has been previously described in detail,^21^ is a custom instrument developed by Nikon, as shown in Fig. 3(a). The microscope is capable of imaging over five wavelengths: 405, 442, 473, 532, and 632 nm, a 5× magnification objective was used in this study, with a 3 mm field of view. The detection system of the polarimeter uses a pair of Savart plates to encode the four Stokes parameters into frequencies in each image taken, and an example of this can be shown in Fig. 3(b). The input illumination is provided by light emitting diodes (LEDs) with a relatively narrow bandwidth, approximately 3  nm.^21^ The polarimeter uses a linear polarizer and a quarter wave plate, which generate six polarization states to illuminate the sample in sequence as laid out in Fig. 3(c). The system automatically cycles through each illumination state and takes an image at each wavelength, resulting in a set of 30 images, as shown in Fig. 3(d). In custom software, also developed by Nikon, these images are then processed by decoding the Savart plate induced interference frequencies to get the outgoing Stokes parameters of the light scattered from the sample. A data reduction algorithm is then applied, which calculates the least-squares optimized Mueller matrix for each pixel at each imaging wavelength. The same software then calibrates out the Mueller matrix responses of the microscope optics and illumination system to isolate the Mueller matrix from the sample. To minimize inaccuracies in the resulting Mueller matrix, this reconstruction can not be applied to certain data sets due to oversaturation, excessive noise or numerical instability. In these cases, the Mueller matrix is not derived for the affected wavelengths, and there are some instances of this in the data below where those data points are omitted.

### 3.3 Data Analysis

Once the Mueller Matrices of the sample are derived, a Lu–Chipman decomposition^51^ is applied using the pySCATMECH library in Python, as shown in Fig. 3(e). This results in separate pixelwise maps for the different polarization properties, including retardance, diattenuation, and depolarization, an example of this is shown in Fig. 3(f). From here, the different polarization properties of tissue regions can be further analyzed. In this case a two-dimensional Gaussian filter with a standard deviation of 10 pixels was applied to the data to reduce noise, and ROI masks were drawn to observe polarization properties of select regions. For the crossing fiber phantoms and optic chiasm, the normalized distribution of retardance angle and diattenuation angle were compared for regions corresponding to crossing fibers and coherent unidirectional fibers, as shown in the following sections as radial plots in which the maximum radial value is the angular value with this highest occurrence in that region. Circular colormaps are provided for spatial retardance angle visualizations, which indicate the color corresponding to the angle of a line tangent of the circle at any given point around its circumference. For the inclined fibers and CST, the retardance and diattenuation values were averaged at each point along the length of the sample at each inclination angle and the mean value was taken over the whole ROI, which could be compared across the different tilt values. With the fibers curving out of plane and the cerebellum, the retardance and diattenuation values were averaged at each point along the fiber and the ridge of the cerebellum, and linear fitting was applied. Finally, for the fibers curving in plane, the distribution of the retardance angle and diattenuation angle were compared to the fiber orientation in several ROIs along the curve, also shown as radial plots made up of the normalized angular values like the crossing fiber data.

### 3.4 Phase Unwrapping

**Fig. 4:**
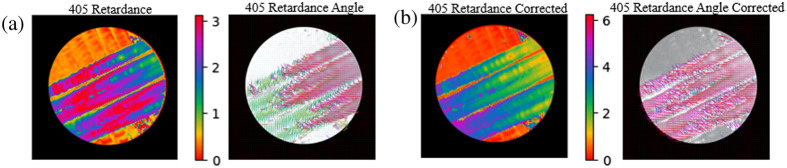
(a) Phase wrapping artifact depicted in retardance and retardance angle data. (b) Corrected retardance and retardance angle plots via phase unwrapping.

Frequently in the polarimeter retardance data, there are large discrepancies both between imaging wavelengths and within individual wavelength datasets, such as in the mirroring of the sign of trends observed around π and a 90-deg offset in the direction of retardance angle. This has been observed in work by other groups working with Mueller matrix polarimetry as a result of a phase wrapping artifact.^52^^–^^54^ Depicted in Fig. 4(a), a retardance plot in which we expect to see the retardance changing monotonically along the length of the fibers and a corresponding retardance angle plot in which we expect the retardance angle to be consistent along the length of the fiber, but we instead see a 90-deg discontinuity. This is a result of Mueller matrix decomposition algorithms, such as the Lu–Chipman, which was applied in this study, in which the retardance is calculated by applying an inverse cosine function to several of the Mueller matrix elements, described in detail by Ghosh et al.^33^

$$R=\cos^{-1}[\frac{tr(M_{R})}{2}-1],$$ (5)

where $M_{R}$ is the retardance Mueller matrix as calculated in the Lu–Chipman decomposition. The inverse cosine function is intrinsically bounded from zero to π radians, but as the Mueller matrix components corresponding to retardance continue to increase past what would be π radians the output retardance is mirrored around π due to the even nature of the cosine function.^52^

It can be somewhat difficult to discern in retardance data; however, this phase wrapping also affects the calculation of the retardance axis of the material, resulting in a 90-deg offset in the retardance angle data, as shown clearly in Fig. 4(a). To correct this, we can rely on the assumption that the phantoms used here are uniaxial and use these discontinuities in the retardance angle as a mask to correct the retardance and retardance angle as shown in Fig. 4(b). To do so, we take what we believe to be the affected portion of the signal and either subtract it from 2π if it is mirrored around π or make it negative if it is mirrored around zero; additionally, we can apply a correction to the retardance angle data by adding 90-deg to the offset angular portions of the data, similar to the methods laid out in Song et al.^52^ This correction clarifies some ambiguities in the data; however, there is a subjective nature to them in determining which parts of the data have undergone phase wrapping due to the relative nature of the retardance measurements. In this work, we mainly relied on the convention that we expect to see a reduction in retardance as wavelength increases, as predicted by our theoretical model. We have applied this phase unwrapping correction to several of the datasets in this study and have noted where this occurs in the text that follows.

### 3.5 Additional Imaging and Processing for Validation

To better interpret the resulting PLI data, additional imaging methods were used for validation. In this case, a spectral domain optical coherence tomography (OCT) system (Thorlabs, TEL221C1) was used to gain structural information about bulk tissue imaging with an LSM04 lens. The OCT data were used to find the radius of curvature of the cerebellum surface at specified ROIs. To do so the two-dimensional cross-sectional ROI was taken, and the radius was found by applying a singular value decomposition based Taubin circle fit.^55^

Additionally, histological staining was applied to the hippocampus cross-section in accordance with methods previously applied on brain tissue.^56^ Cellular membrane staining was accomplished using 1,1’-dioctadecyl-3,3,3’,3’-tetramethylindocarbocyanine perchlorate (DiI) where slides were deparaffinized in a 100% xylene bath for 25 min and rinsed in ethanol for 20 min. The 0.25  mg/ml DiI stain was prepared in a 100% ethanol solution where slides were submerged for 10 s to initiate membrane staining. Finally, slides were submerged for 10 minutes at a time to initiate rehydration in a graded ethanol series ranging from 95-0%. Coverslipping was done with Vectashield, antifade mounting media. The stained sample was then imaged by a slide scanner. In ImageJ, the RGB image obtained in brightfield imaging was converted to an 8-bit image. OrientationJ, an ImageJ plugin, was used to characterize the orientation and isotropic properties of fibers. OrientationJ was applied using a structure tensor having a gaussian window of 2 pixels and a cubic spline gradient algorithm where the hue corresponds to orientation, the color saturation corresponds to the fiber coherency, and the brightness corresponds to the original 8-bit image.

## 4 Results and Discussion

### 4.1 Crossing Fibers

**Fig. 5:**
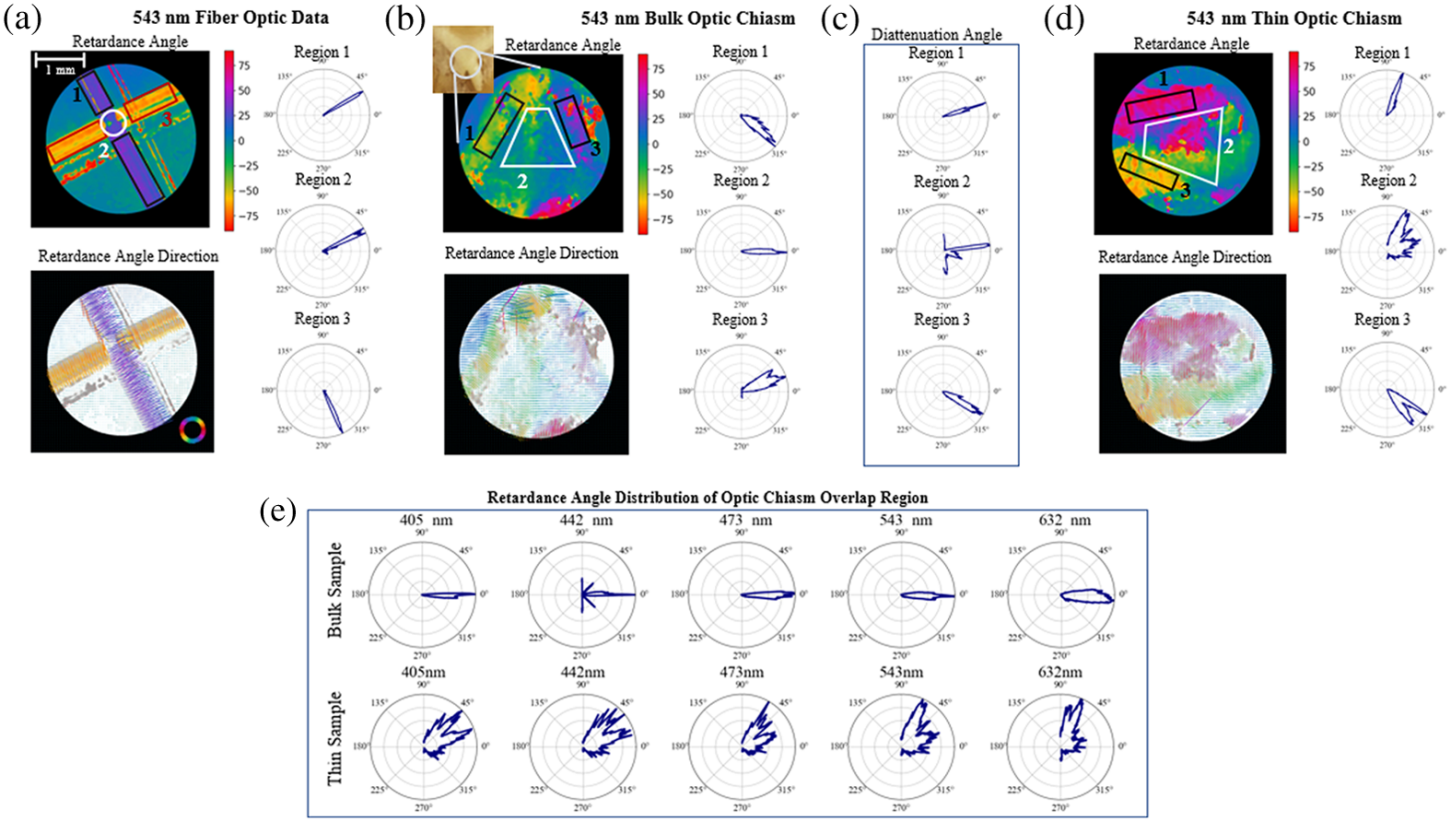
(a) Crossing fiber optic retardance angle data with angular distribution of retardance angle shown for the top fiber, bottom fiber, and crossing fiber region on the right. (b) Bulk optic chiasm retardance angle data with angular distributions of the upper region, lower region, and crossing fiber region shown on the right. (c) Optic chiasm diattenuation angle distributions of the upper region, lower region, and crossing fiber region. (d) Thin, 7  μm thick, optic chiasm slice retardance angle data with angular distributions of the upper region, lower region and crossing fiber region shown on the right. (e) Optic chiasm retardance angle data distributions within the crossing fiber region shown across five different imaging wavelengths for bulk sample (top row) and thin sectioned sample (bottom row). For the angular data shown here: 0 deg is aligned horizontally, pointing to the right in (a) and (d), and vertically pointing down for (b) and (c). Positive are measured counterclockwise.

To examine the PLI system’s response to crossing fibers the directional data was analyzed—namely the retardance angle for the fiber optics and synthetic hair and diattenuation angle for the monofilament thread. Comparing the data outside the crossing fiber regions and inside the crossing fiber region, the fiber that is on top in the region of crossing fibers is the dominant source of signal for all three phantom types with very little detectable signal from the underlying fiber. An example of this for the fiber optic cable imaged at 543 nm is shown in Fig. 5(a), in the crossing fiber region the retardance angle distribution clearly aligns with that of the top fiber. The retardance axis is aligned perpendicular to the overall fiber direction, which is consistent with a negatively birefringent material.

Comparing this to the bulk optic chiasm data shown in Fig. 5(b), in the crossing fiber region the retardance angle goes in one direction, which is approximately the average of the two edge regions. The bulk optic chiasm diattenuation angle data, as shown in Fig. 5(c), has a broader angular range than the retardance angle data in the region of crossing fibers; however, it seems to be more in agreement with the phantom data in that the direction of the dominant angle more closely aligns with the dominant angle from the left side of the chiasm, region 1 in Fig. 5(b).

Comparing the bulk optic chiasm data to a thin sample sectioned shown in Fig. 5(d), the retardance signal of the thin sample results looks more similar to the phantoms in that the crossing fiber region is much more closely aligned with one of the edges rather than an average of the two. The thin sample was imaged slightly further up the optic chiasm with a 90-deg rotation from the bulk sample’s orientation.

Considering the effects of illumination wavelength, at lower wavelengths, one fiber direction dominates much of the retardance angle signal; however, as the wavelength of the illumination light increases, there is a slight spread over a broader range of angles between the two dominant angles of the edge regions. The change in angular distribution for the retardance angle data with wavelength is laid out in the top row of Fig. 5(e). This could be due to increased depth penetration of the light as the wavelength increases or increased noise seen at higher wavelengths.^21^ For the thinly sliced optic chiasm, Fig. 5(e) (bottom row), we see the retardance angle is more broadly distributed at lower wavelengths and then starts to favor one direction at the higher wavelengths. This is the opposite of what we see with the bulk sample, and in this case, the differences cannot be attributed to different penetration depths of the light. The sectioned sample thickness is 7  μm, which is well within the penetration depth of light into tissue—around 0.5 mm for the lower wavelengths and 1.5 mm for higher wavelengths.^57^ The broader distribution of angles at shorter wavelengths could be instead due to higher scattering rates for the shorter wavelengths at a constant depth.

The phantom fiber and thin sectioned sample data seem to be in agreement with previous work simulating and measuring the effects of crossing fibers in transmission PLI systems,^58^ which found that crossing fibers would create a patchwork effect, implying which ever fiber is on top of the crossing dominates the signal. Alternatively, a recent study with this same microscope system found evidence to suggest that the retardance angle data would be averaged over in regions of crossing fibers,^45^ which is a more accurate description of the bulk optic chiasm retardance angle data. These changes in measurement response could also come down to resolution, the diameter of the phantom fibers used here is much larger than the resolution limit of the microscope—10 microns,^21^ where the neural fibers will be in actuality much finer and thinner potentially resulting in averaging effects in regions where the fibers cannot be individually resolved.

### 4.2 Inclined Fibers

**Fig. 6:**
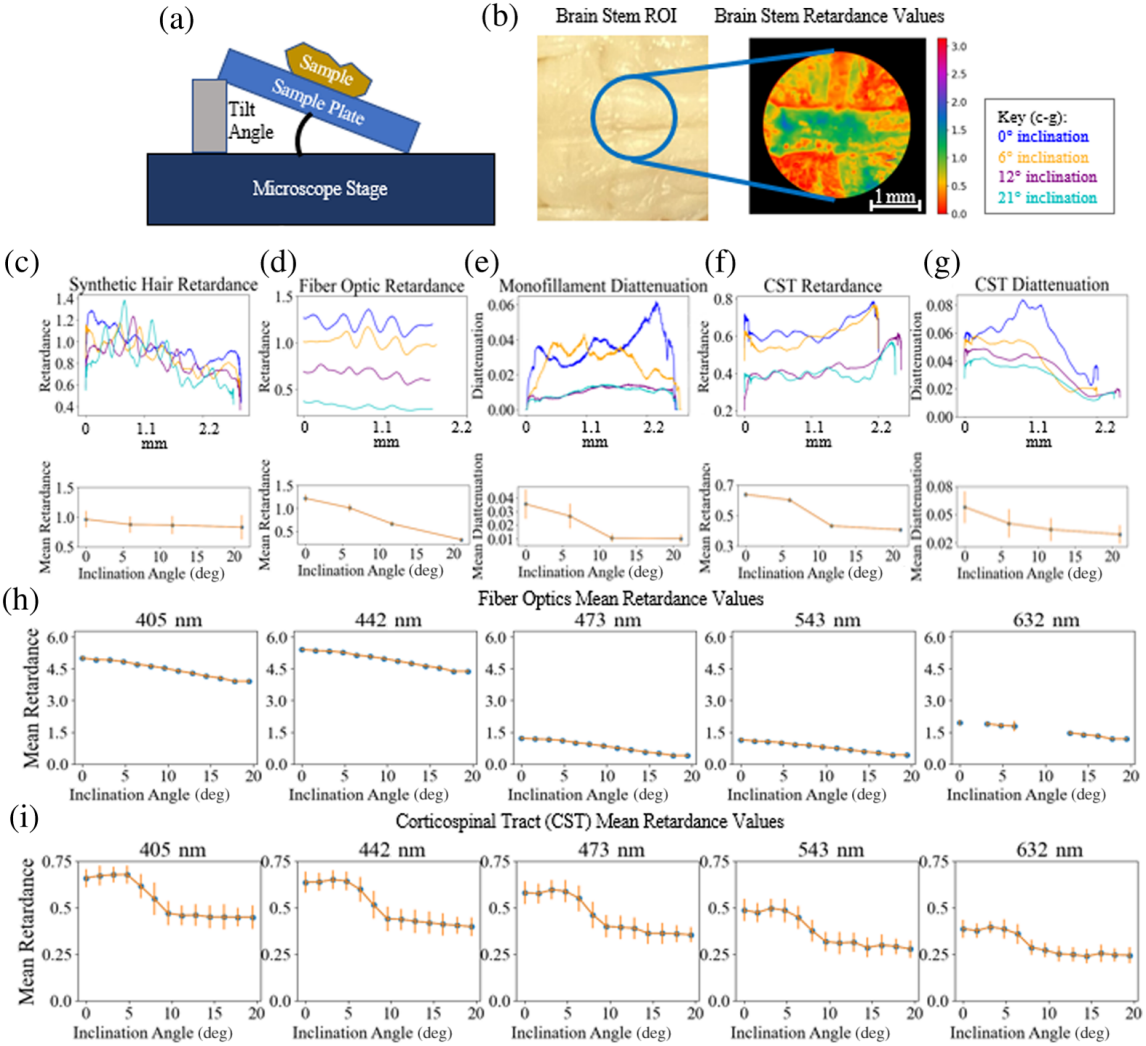
(a) Inclination configuration for brain tissue and fibers. (b) Corticospinal tract (CST) ROI with retardance data from that region shown imaged at 543 nm. Above, retardance values taken along the length of the synthetic hairs (c) and fiber optics (d) overlaid for each inclination angle, mean retardance value of the fibers versus angle shown below. (e) Diattenuation values taken along the monofilament threads overlaid for each inclination angle shown above, mean diattenuation value of the monofilament thread versus angle shown below. On top, retardance (f) and diattenuation (g) values taken along the length of the CST are overlaid for each inclination angle. Below, mean values are plotted from each CST ROI inclination angle data set for retardance (f) and diattenuation (g). (h) Mean retardance values for fiber optics over finer sampling of inclination angles at the five different imaging wavelengths, phase unwrapping applied here. Data points missing in the 632 nm results because the Mueller matrix data reduction could not be applied. (i) Mean retardance values for CST data over finer sampling of inclination angles at the five different imaging wavelengths.

To assess the polarimeter’s sensitivity to inclination angle three of each type of the phantom fibers were secured to a plate, which was lifted up at several intervals, as shown in Fig. 6(a). This was then repeated on the ferret brain sample to image the CST [Fig. 6(b)]. There is a very slight downward trend visible with increase in inclination angle for the synthetic hair data [Fig. 6(c)] and a more prominent downward trend with the fiber optic data [Fig. 6(d)]. We would expect with the theoretical model laid out in Sec. 2 that the synthetic hair retardance values would increase with inclination angle given our assumption that the hairs are positively birefringent. This may suggest our assumption about the birefringence of the material was flawed. However, it is worth noting that the standard deviation of each data set is larger than the differences in retardance values observed with tilt, so perhaps the divergence from our model could be due to some of the other factors laid out in Sec. 2, such as the influence of imaging wavelength or path length through the material. While the data shown here were imaged with 442 nm light, there is an upward trend in the 632 nm data, which suggests the influence of wavelength may outweigh the influence of tilt in this case.

The diattenuation values were taken across the monofilament fiber, as shown in Fig. 6(e). The mean diattenuation values decrease with increase in inclination angle. In comparison to the ferret brain data, the retardance values for the CST, as shown in Fig. 6(f) imaged at 442 nm, and the diattenuation values, as shown in Fig. 6(g), also decrease with increase in inclination angle.

Overall, the noise in the data threatens to eclipse differences that may emerge from inclination angle, especially in the fibers with weaker signal. This effect is pronounced in the diattenuation data which is much smaller in magnitude than the retardance values, limiting the confidence with which conclusions can be made from it. The data shown here has had Gaussian smoothing applied to better visualize differences between the inclination angles. In considering the effects of noise over different wavelengths: in general, with the fiber phantoms, the signal was noisier with higher wavelengths; in imaging brain tissue the noise level was relatively consistent across all wavelengths but the retardance signal was slightly weaker with higher wavelengths making the noise present more pronounced.

To further explore the effects of inclination angle on retardance signal, the fiber optics and CST were re-imaged with smaller differences in inclination, and the resulting means of retardance data as a function of inclination angle are shown in Fig. 6(h) for the fiber optics with phase unwrapping applied and Fig. 6(i) for the CST. There are some missing data points in the 632 nm data, for these datasets the Mueller matrix data reduction could not be applied due to instabilities in the data.

One limitation of using the fiber optics as phantoms is the stronger retardance signal introduces the need for phase unwrapping, in comparison to the CST results for which the retardance is less than one and decreases with inclination angle across all wavelengths. Before unwrapping the fiber optic retardance varies across the range of zero to π across all datasets. Use of thinner fibers in the future could be one method for reducing the necessity of phase unwrapping.

The CST sample also shows a fairly consistent pattern as inclination angle increases, with an initial increase in mean value then a decrease and leveling off. This diverges somewhat from the model laid out in Sec. 2; while there is an overall decrease with increase in inclination angle and across the different imaging wavelengths, the general shape of the curve does not align well. The leveling off that occurs at higher inclination angles may be due to lack of system sensitivity and difficulty focusing the microscope on tissue that is beyond the depth of focus. However, these divergences from the model are not observed in the phantom data, which was imaged with the same angular inclination. This suggests there are aspects of the tissue contributing to the retardance measurements which have not yet been fully factored in.

### 4.3 Curving Fibers

**Fig. 7:**
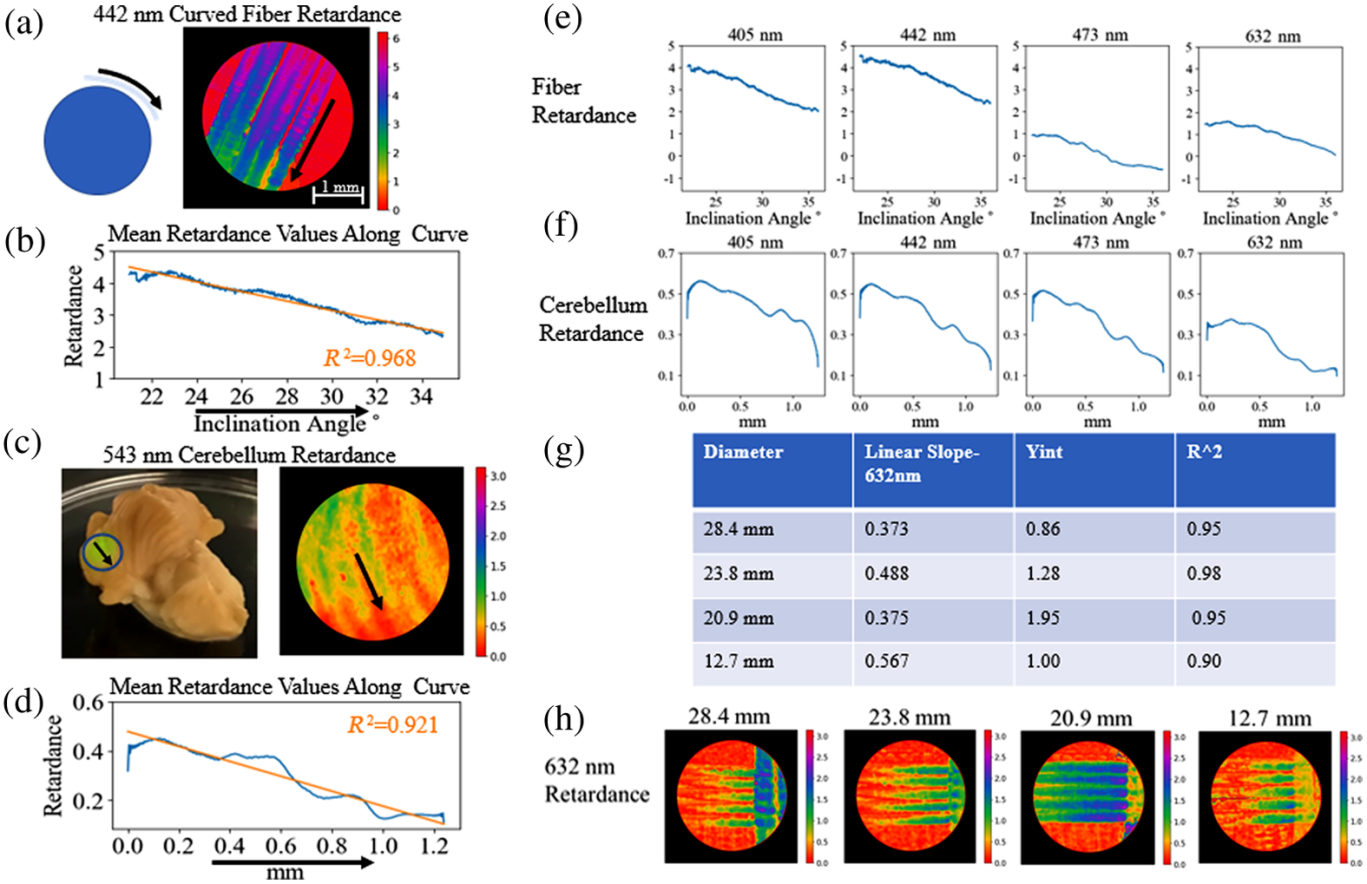
Phase unwrapping algorithm applied to curved fiber data in this figure. (a) Fiber optic imaged over a 28.4 mm cylindrical form to assess the effects of curvature on retardance, retardance values imaged at 442 nm shown. (b) Mean retardance values along the fibers imaged in a. with the arrow indicating downward direction. (c) Cerebellum region imaged with retardance values at 543 nm shown. (d) Mean retardance values along one of the ridges of the cerebellum with the arrow indicating downward direction. (e) Mean retardance values along the fibers imaged in a. at 4 different imaging wavelengths. (f) Mean retardance values along the one of the ridges of the cerebellum at four different imaging wavelengths. (g) Linear slope of retardance values, and y intercept of fibers curving out of plane with different radii of curvature. (h) Retardance of fibers curving out of plane with different radii of curvature.

To continue to examine the effects of fiber inclination, we imaged the fiber optic phantoms curved over a cylindrical form, as shown in Fig. 7(a). Once again in agreement with the model laid out in Sec. 2, when the mean retardance along the curve was taken there seemed to be a nearly linear decrease in retardance as the downward slope of the fiber increased, as shown in the data in Fig. 7(b), which has had phase unwrapping applied to it. While the model is not linear, this data is taken over a smaller range of angles so we are able to approximate it with a linear fit. This can be compared to the changes in retardance values across downward sloping regions of the cerebellum, as seen in Fig. 7(c). Along the ridges of the cerebellum, we expect there to be a series of parallel fibers,^59^ which have been shown to induce a retardance signal.^45^ The retardance along the downward curve of the cerebellum exhibits characteristics similar to what is observed with the phantom, in that retardance signal decreases along the curve. There are some larger deviations from a linear fit, as shown in Fig. 7(d), which could be a characteristic on the less homogeneous and isotropic sample.

In Fig. 7(e), the retardance signal across the different imaging wavelengths can be viewed after phase unwrapping has been applied, and in this case, the retardance undergoes a decrease from the lower toward the higher wavelengths but not monotonically. In this case, the 543 nm data was ommitted due to errors in the Mueller matrix data reduction algorithm. Once again, the directional trend of the tissue data remains consistent across the five wavelengths, as shown in Fig. 7(f). This supports the results shown earlier in Fig. 6 with CST inclination.

In an attempt to investigate how the radius of curvature changes the trend in retardance, the fibers were laid over cylinders with different radii and imaged. As shown in Fig. 7(g), while an obvious trend in relating the linear slope to radius of curvature does not emerge from this data, there are different slopes and intersects of the linear fit, which can be visualized in Fig. 7(h).

**Fig. 8:**
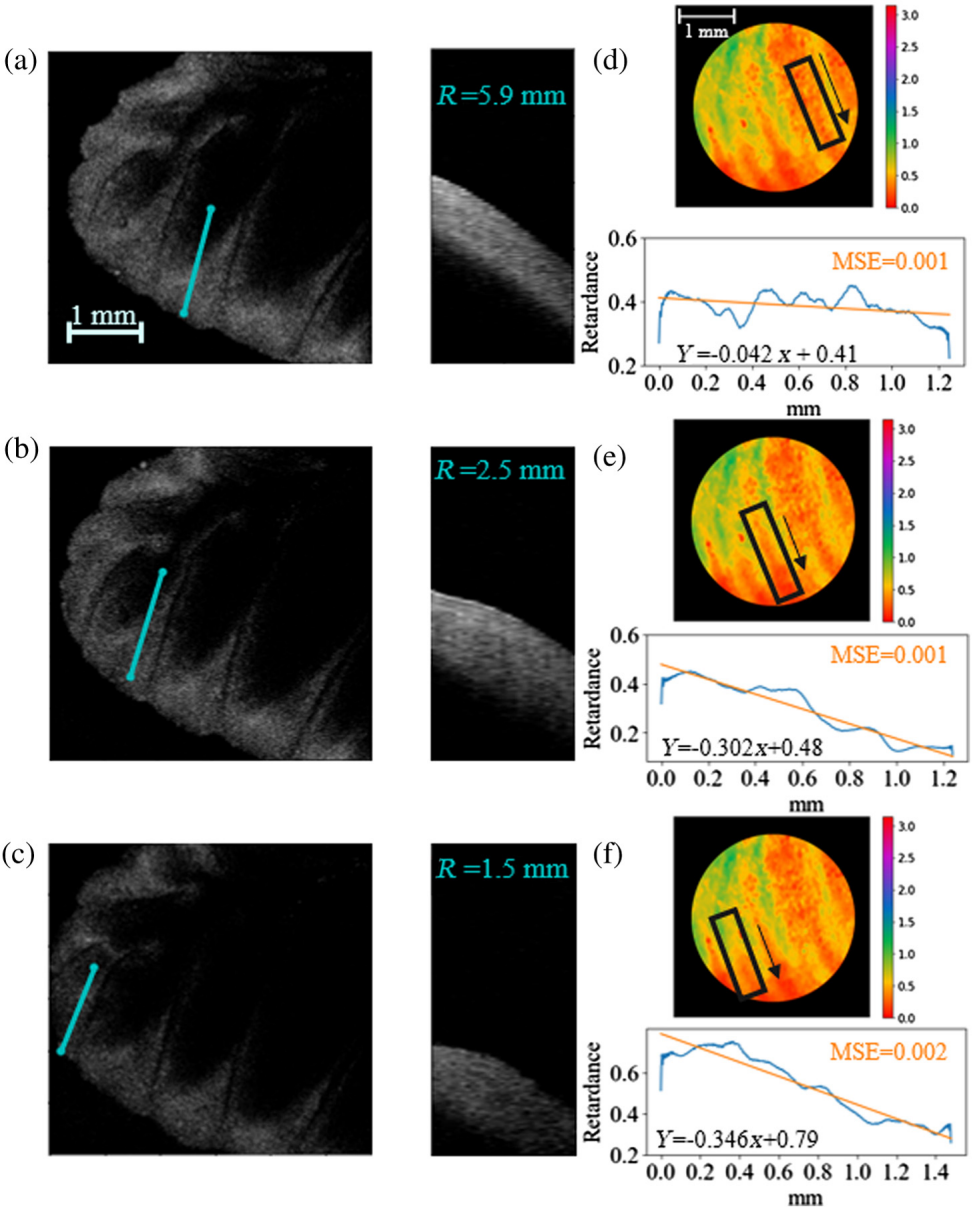
(a)–(c) Images of cerebellum taken with OCT shown from above on the left, cross-sectional region taken along the indicated blue line with the approximated radius of curvature of the cross section noted on the right. (d)–(f) Corresponding cerebellum regions imaged with the PLI system with mean retardance values taken along similar lines for comparison.

To examine the effects of radius of surface curvature on retardance values in brain tissue, different ROIs were selected from PLI data taken of the cerebellum. The radii of curvature of these regions were determined by imaging the sample with an OCT system. As shown in Fig. 8, three different cross-sectional regions with different radii of curvature, Figs. 8(a)–8(c), can be directly compared to retardance values along those curves in Figs. 8(d)–8(f). In this case, the slopes and y-intercepts of the three linear fits increase as the radii of curvature of the region decreases. It is necessary to note that these are not perfect cylindrical curves, which could contribute to this deviation as well as tissue inhomogeneity. Despite this, these results suggest a relationship that could be a valuable avenue of future study.

### 4.4 Effects of Rotating Tissue Samples

There have been previous studies, which have utilized rotation in polarimetry systems to glean information about the inclination angle of the fibers within a tissue sample.^6^^,^^13^ In this method, a rotating polarimeter is used to image a sample at a range of discrete angular orientations. From here, the inclination angle of the fibers can be determined by isolating changes in measured intensity, which relate orientation angle of the system to retardance, which has a dependence on the inclination of the fibers being measured.^6^ This method has not yet been broadly applied in reflection-mode PLI systems, so to consider the feasibility of this we test the effects of rotating a sample on the microscope stage.

**Fig. 9:**
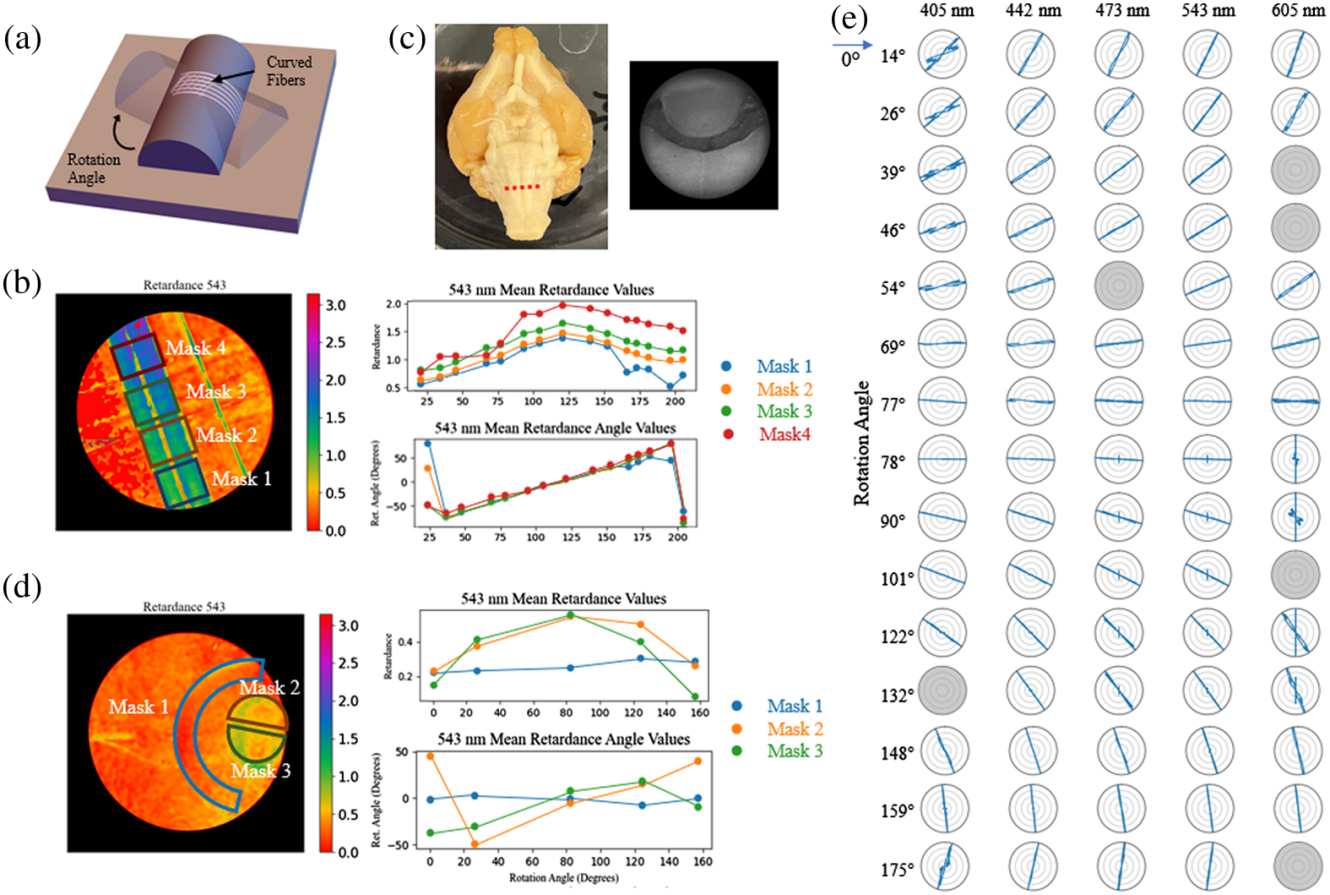
(a) Rotation configuration for curving fibers. (b) Mean retardance and retardance angle values from four different ROIs, noted on the retardance map on the left, of the curving fibers plotted against orientation angle. (c) Ferret brain ROI, with region the section was taken from shown on the left with red dotted line. (d) Mean retardance and retardance angle values from three different ROIs, noted on the retardance map on the left, of a cross-sectional region of the brain stem plotted against orientation angle. (e) Retardance angle values taken of fiber optics imaged over a cylindrical form at 15 different rotation angles across the five different imaging wavelengths, with greyed out plots denoting data sets for which the Mueller matrix data reduction could not be completed. A 90 deg rotation has been applied to the 405 and 442 nm data to account for phase wrapping.

First, working with our fiber optic phantom, we rotated the fibers curving out of plane over 15 different orientation angles, an example of this is shown in Fig. 9(a). The rotated datasets are then co-registered to one another and ROI masks are drawn at different points along the curve to simulate the effects of rotation of different fiber inclination angles. As shown in Fig. 9(b), the retardance values change with the orientation angle of the phantom in a somewhat sinusoidal pattern, with a vertical offset of the data for each region of interest. In comparison, the retardance angle values change linearly with change in angle, which is to be expected.

Comparing this to a cross-sectional region of brain stem, Fig. 9(c), once again the sample is rotated and the datasets are co-registered to one another. With this we are able to look at three different regions, two with a strong retardance signal, masks 2 and 3, and one without, mask 1, across the different rotation orientations. In looking at the retardance values, Fig. 9(d), we see a similar sinusoidal trend emerging as we see with the phantoms in the two regions with a strong retardance signal. The trend in retardance angle values is less clearly linear than those from the phantom data, but there is a somewhat linear trend in the data from masks 2 and 3, aside from a few outliers.

In considering the relationship between these trends and the neural microstructure: it is known that the fibers in mask 3 region of the brain data are coming out of the plane. The reduced retardance in this region is consistent to what we saw in Sec. 4.2 with inclined fibers. The microstructure in the masks 2 and 3 regions is not precisely known due to this being a heterogeneous region of the brain with varying fiber directions depending on precisely where this section was taken from.

While this brain stem data is relatively preliminary, the clarity of the trends seen in the phantom data and resemblance we see in the brain data suggest this is worth further investigation. Future steps include sectioning and staining this region to determine the precise orientation of the fibers.

Beyond the potential effects of fiber inclination, this phantom study is also useful in determining the sensitivity of the polarimeter to changes in orientation angle. The resulting retardance angle distributions for the five different imaging wavelengths are shown in Fig. 9(e), these show the retardance angle distribution seems to be providing an accurate directional response when we have a strong retardance signal, such as those from these fibers. The angular direction is offset by approximately 90  deg from the fiber’s orientation, which is in agreement with our assumption that these fibers are negatively birefringent. In Fig. 9(e), some of the polar plots are grayed out. This is due to instabilities in those data sets that made the Mueller matrix data reduction infeasible. Also, the 405 and 442 nm data have had a 90 deg rotation applied to the angle because phase wrapping was observed in those datasets.

### 4.5 Curving Fibers In-Plane

**Fig. 10:**
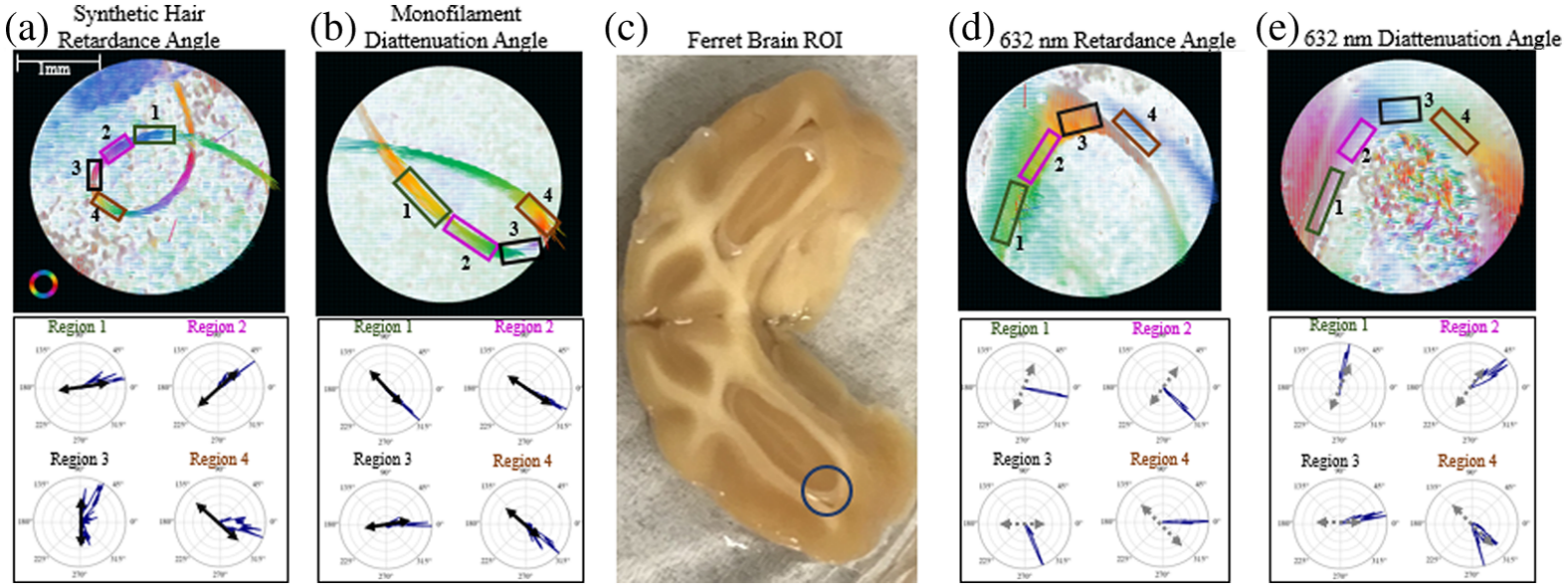
(a) Retardance angle map scaled by retardance of the synthetic hair curving in the plane of the microscope stage with angular distributions of selected regions shown. The dominant fiber direction in each region is overlaid on each in black. Scale bar shown in (a) also applies to (b), (d), and (e). (b) Diattenuation angle map scaled by diattenuation of the synthetic hair curving in the plane of the microscope stage with angular distributions of selected regions shown below. The dominant fiber direction in each region is overlaid on each in black. (c) Ferret brain cross section with imaged cerebral white matter region circled. (d) Retardance angle map distributions of the cerebral white matter region scaled by retardance with angular distributions of selected regions shown below. The suggested dominant fiber direction in each region is overlaid on each in gray. (e) Diattenuation angle map of the cerebral white matter region scaled by diattenuation with angular distributions of selected regions shown below. The suggested dominant fiber direction in each region is overlaid on each in gray. For the angular data shown here: 0 deg is aligned horizontally, pointing to the right, and positive angles are measured counterclockwise.

To investigate the response of the retardance and diattenuation angle direction to in-plane curvature, a synthetic hair and monofilament thread were secured in loop configurations and laid flat on the microscope stage to be imaged. In Fig. 10(a), the resulting retardance angle map is shown, with the angular distributions of selected regions shown below. The retardance angle closely follows the direction of curvature of the hair. This is in agreement with our previous assumption, that the hair is positively birefringent—in that the retardance axis of the fiber would be along its length.

The diattenuation angle of the monofilament fiber, as shown in Fig. 10(b), shows similar results in that the diattenuation angle closely follows the orientation of the fiber. This suggests that the diattenuation angle may be a useful tool in determining microstructural orientation in regions with strong diattenuation signal.

In comparison to the curvature in the cerebral white matter region imaged here, as shown in Fig. 10(c), we are able to detect orientation information from both the retardance angle [Fig. 10(d)] and the diattenuation angle [Fig. 10(e)]. In observing these results, it is important to note that the precise fiber orientations of this cerebral white matter region are unknown and the directions selected here are the visually observed paths of overall structural orientation. In this case the diattenuation angle follows the apparent direction of the fiber curvature closely while the retardance angle seems to be approximately perpendicular to the orientation direction. This 90 deg offset is to be expected as white matter fiber tracts are known to be negatively birefringent.^13^ There are some divergences from that perpendicularity in regions 3 and 4, as shown on the directional plots in the lower half of Fig. 10(d). This may be because there could be unknown fiber orientations in those regions where the retardance is more sensitive than the diattenuation.

**Fig. 11:**
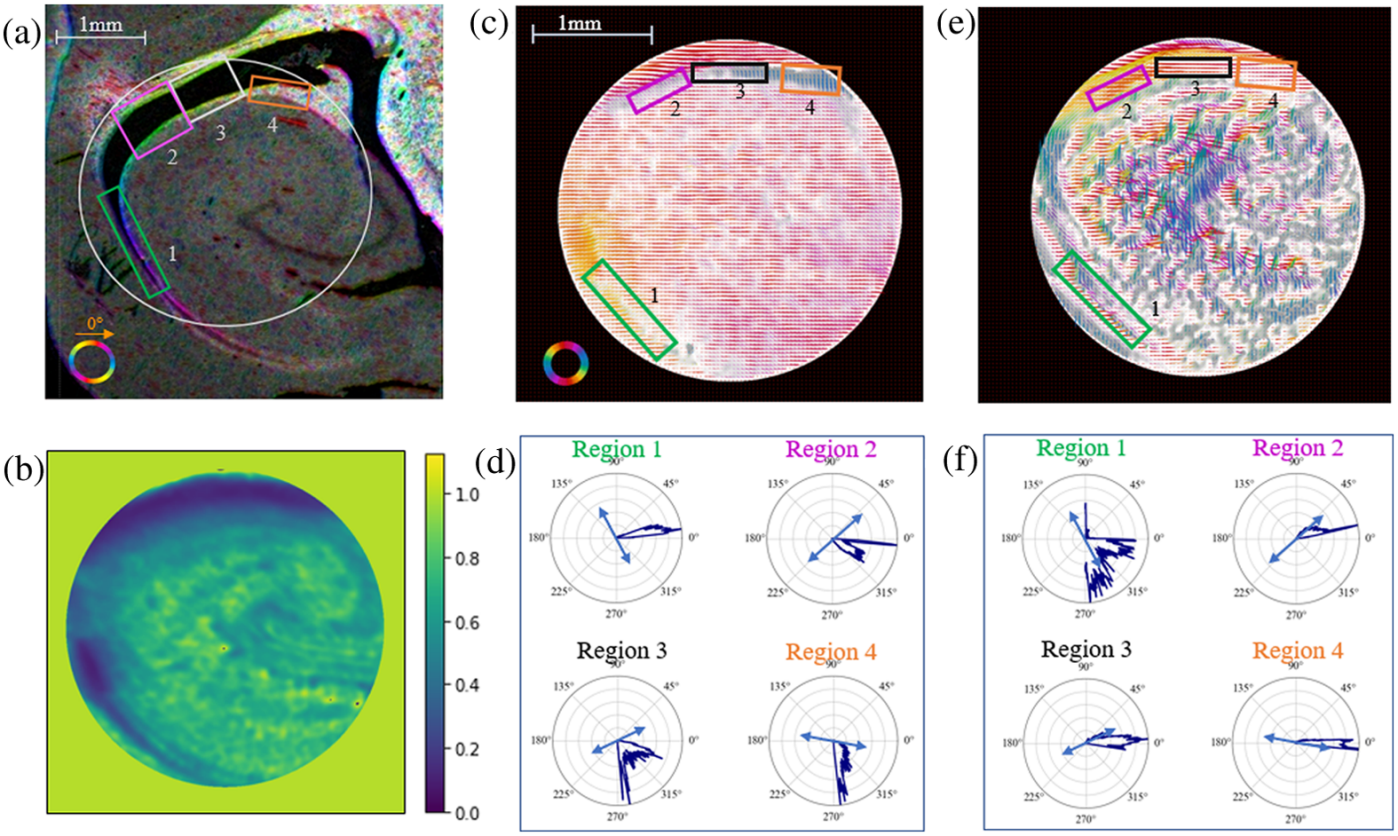
(a) DiI stained hippocampus slide imaged with a slide microscope and processed with orientationj in imagej. (b) Depolarization PLI data of hippocampus region. (c) Retardance angle PLI data of hippocampus region scaled by retardance. (d) Retardance angle distributions of selected regions shown with dominant direction indicated by orientationj overlaid in a blue arrow. (e) Diattenuation angle PLI data of hippocampus region scaled by diattenuation. (f) Diattenuation angle distributions of selected regions shown with dominant direction indicated by orientationj overlaid in a blue arrow. For the angular data shown here: 0 deg is aligned horizontally, pointing to the right, and positive angles are measured counterclockwise.

To validate orientation features in white matter, we compare PLI results to those of a diI stained and thinly sectioned hippocampus sample form our cohort of ferret brains, as shown in Fig. 11(a). A region of nearby hippocampus was also measured with the PLI system, with the measured depolarization shown in Fig. 11(b). The PLI retardance angle data scaled by the retardance [Fig. 11(c)] and the angular distributions of select regions, shown in Fig. 11(d), can be directly compared to the directional information derived from the diI sample. This seems to show a similar sensitivity to the directionality of the white matter surrounding the hippocampus region as the cerebral white matter region in Fig. 10, in that once again the dominant retardance angle signal is approximately perpendicular to the indicated direction of the diI sectioned slide, with a wider spread of angular data in region 3, which has a lower retardance signal. This is consistent with our observations in Fig. 10, and again shows the effect of the negatively birefringent white matter. The diattenuation angle data, as shown in Figs. 11(e) and 11(f), once again follows the orientation direction closely, aside from in region 1, which has a wider spread of angular distribution, perhaps due to lower diattenuation signal in that region.

Overall, these results suggest that this PLI system is sensitive to fiber orientation and shows promise for the use of microstructural mapping for both the retardance angle and diattenuation angle data in regions with strong enough signal.

## 5 Conclusion

This study allowed for quantitative assessment of several parameters that have not yet been thoroughly investigated for backscattering polarimetry systems, including fiber inclination angle, the rotation of fibers curving out of plane, and the comparison of imaging across five different wavelengths. Easy to manipulate polarization phantoms were used, which allow for direct comparisons to relevant brain tissue regions of similar fiber architecture. While all these factors have been studied to some degree previously, this work provides an analysis of how the combined effects of these factors manifest in the retardance, retardance angle, diattenuation, and diattenuation angle data.

From this, data observations can be made about the polarimeter’s response and sensitivity to crossing fibers, fiber inclination and curving fibers. We were able to conclude that in regions of crossing fibers the top fiber dominates the signal in the phantom and thinly sectioned sample, however in the bulk tissue sample we see averaging effects of the two edge fiber directions in the crossing region. For the fiber inclination, we see reduced retardance and diattenuation with increased tilt angle. This effect is also seen with fibers curving out of the plane, with the slope of the decrease showing some correlation to the radius of curvature for the cerebellum tissue. When these fibers are then rotated in the imaging plane, we see changes in the retardance values, which could be indicative of some directional sensitivity to the system’s response to out of plane fibers. The phantoms also allow us to study the sensitivity of the system to in-plane curvature of fibers, which we find to be traceable with both the diattenuation angle and retardance angle.

Further study of each of these areas will aid in assessing the potential of this system for characterizing the response to certain tissue features. Future steps include a more complete investigation of the effects of imaging at different wavelengths. Additionally, the question arises as to whether full Mueller polarimetry is necessary for a given application. While acquiring the Mueller matrix of a sample provides the most complete picture of its polarization properties, in brain imaging applications, it seems that retardance and depolarization are the most prominent polarization properties.^12^^,^^16^ Diattenuation has been examined to some extent in the context of transmission PLI;^60^ however, in general, the magnitude of diattenuation is significantly lower in tissues compared to retardance and depolarization, which may inhibit precise measurements, lending to its infrequent use.^7^ However, the resulting data from this study suggests that diattenuation angle may also be a useful metric in mapping fiber direction. These investigations will be made with the consideration of potential for *in vivo* imaging to better understand the capabilities and limitations of not only this polarimeter but backscattering configured polarimeters in general.

## Data Availability

The data and materials used in this study can be made available upon request via the corresponding author.
